# Medical Items and News of Interest to the Physician

**Published:** 1875-10

**Authors:** 


### Medical Items and News,

### For the Use of Physicians.

CULLED FROM THE STANDARD MEDICAL JOURNALS OF THE WORLD.

**NOTE.—These formulæ are intended only for the regular practitioner of medicine, and should be employed only by him, or under his immediate supervision.**

### Local Anæsthetic.

Pulv. camphoræ, ʒ iiss.

Ætheris sulph., ʒ v. M.

Rub into the skin for a few moments at the locality where it is desired to produce anæsthesia.—*Med. Times.*

### Rheumatism.

℞

Trimethylamini, ♏ iv ad ♏ viij.

Syr. zingiberis, ʒ i.

Aq. menth. pip., ℥ i. M.

Take at intervals of one to three hours until pain is relieved.—*Med. Times.*

### To Remove India-Ink Marks.

Rub well with a salve of pure acetic acid and lard, then with a solution of potash, and finally with hydrochloric acid. Sometimes these marks may be obliterated by blistering the skin and keeping the blister open for a little while. When the new skin grows, the marks will have disappeared.

### Antidote to Carbolic Acid.

A writer in the Dublin *Medical Journal* states that a true antidote to carbolic acid remains yet to be discovered, and some recent experiments of M. Gallippe go to discredit the value of saccharate of lime, which has been specially recommended by Kunde, and, from experiments on dogs, he is inclined to place more reliance on olive oil.

### New Method of Administering Raw Meat.

M. Taillier, the head apothecary at the Asylum of Quartre-Mares-Saint-Jon, employs the following preparation for the insane patients to whom it is necessary to administer raw meat: Grated raw meat, 100 parts; powdered sugar, 40 parts; wine, 20 parts; tincture of cinnamon, 3 parts. The sugar is incorporated with the raw meat in a marble mortar, and then the wine and tincture are added. A mixture like marmalade is obtained, having an agreeable flavor, and possessing all the requisites of a tonic and revivifying diet.—*Repertoire de Pharmacie.*

### Carbuncle.

Dr. J. C. Nott, of New York, (*N. Y. Med. Journal,*) advocates the local use of carbolic acid in carbuncle, and publishes an obstinate case which was promptly cured after seven applications. He believes this acid to be more penetrating in its action, more efficient as an antiseptic, than subsulphate of iron.

### Biliary Calculi.

John Barclay, M. D., Physician to Leicester Infirmary, (*British Med. Journal,*) recently gave to a clergyman, aged 58, in his third attack from gall-stones, chloroform in two or three-drop doses, three or four times a day, and to his surprise pain, tenderness, distension, and jaundice disappeared together.

### The Administration of Chloral.

E. Lambert, late House Surgeon of the Maternity Hospital, Edinburgh, (*Edinburgh Medical Journal,*) affirms that the proper mode of exhibiting chloral is in fractional doses of 15 grs. every quarter of an hour until some effect is produced. Some patients will require doses of ʒ j.; and it is better to produce an anæsthetic effect by ʒ iij. given in the space of two hours than by ʒ j. given singly.

### Asthma.

In an interesting discussion on the management of asthma, by the members of the Baltimore Pathological Society, Dr. Garretson (*Baltimore Med. Journal*) alluded to the case of Colonel Washington, subject to attacks of this affection lasting four or five days, who was relieved in two days by bromide of potassium and belladonna.

### Applying a Tampon.

Take a piece of fine rubber cloth,—a piece of soft muslin or a pocket handkerchief will answer, —oil it upon the outside, if you choose, and then, placing one or two fingers in the centre of it, letting the cloth hang over the hand, carry it into the vagina as far as desirable, or whatever cavity you wish to tampon, and can carry the finger. This can be done without giving the patient any more discomfort than does a digital examination. Now we have a collapsed, pouch-like cavity, which can be distended at the pleasure of the operator; may be filled without risk of injuring the soft parts; and without the tugging ordinarily experienced when a tampon is introduced without a speculum.

**Acute Mania and Other Hyperæmic States of the Nervous Centres.**

Dr. Van Andel has employed a solution of ergotin in alcohol and glycerine hypodermically, in the above-named conditions, he states, with satisfactory results. The ergot he believes contracts the cerebral vessels.—*Schmidt's Jarhbucher, February*, 1875.

---

**Warts.**

Lisfranc immerses the parts on which the warts are developed in a strong solution of black soap. This causes a slight cauterization of the surface of the wart. The loosened tissue is to be removed and the application repeated every day till the cure is complete. Oil of vitriol should never be used for this purpose; it is very irritating, and inflames the warts instead of curing them.

---

**Champhor Ointment.**

℞
Pulv. camphoræ, gr. xv;
Glycerinæ, q. s.;
Axungiæ, ℥ i.

Useful in erythema and in vesicular and squamous affections of the skin.—*Medical Times.*

---

**Cystitis.**

℞

Sodii hyposulphit., ℈ iv;

Aq. destillat., lb. i, ℥ iv. M.

This solution may be employed in five injections, in chronic catarrh of the bladder, when there is pain and the carbolic acid injection cannot be employed.—*Medical Times.*

---

**Obstetrical Tincture.**

Long used in abnormal adherences of the placenta, and just published by Dr. Liegard, of Caen, France, who has since died.

Take—Savine, ʒ v.

Rue powd. ʒ ij.

Ergot, ʒ ij; ℥ j.

Uva ursi, ʒ iv.

Alcohol 90 *p. c.*, ℥ v.

Macerate eight days, agitate twice a day, express, filter, and add ergotine, grains xv.

*Mode of Administration*, by teaspoonful in three large spoons of coffee, one a few moments before delivery, a second, twenty minutes after the birth of the child, and a third, half an hour after the expulsion of the placenta.

Three hours later the womb will be found properly retracted, no pains; if any, two additional doses may be given.—*Dr. E. Seguin.*

**Skin-Grafting Superseded.**

Recent statements on the interesting subject of skin-grafting seem to show that they were mistaken who at first thought it necessary to transplant pieces of skin of any depth. All observers now agree that the first phenomenon is the disappearance of the characters of true skin in the piece transplanted, which becomes a mere pellicle. Further, there should be no trace of areolar or adipose tissue left, and Dr. Fiddes says that there is no necessity to take any skin, but merely a few epidermic scales.—*Med. Press and Circular.*

---

**Removal of External Hemorrhoids.**

Dr. John H. Packard, Surgeon to the Episcopal Hospital, Philadelphia, is satisfied that the ecraseur affords the easiest and best means of dealing with external hemorrhoids. He passes two needles, (the curved are the most convenient,) at right angles to one another, through the base of the hemorrhoidal mass, and separates it once. By keeping the bowels confined with opium until the healing is accomplished, the patient suffers no pain, and above all is, relieved by one single procedure; there is nothing left to come away.

---

**Chronic Hydrocephalus.**

Dr. N. S. Davis, Chicago, Ill., (*Chicago Med. Examiner*,) advocates the subjoined prescription in the treatment of chronic hydrocephalus:—

℞
Fl. ex. scuttellaria, ℥ ij.
Tinct. digitalis, ℥ ss.
Iod. potass., ʒ ij.
Fl. ex. hyoscyamus, ℥ ss. M.

Dose, twenty drops, four times a day, in sweetened water. If the digitalis is found to be exerting too much influence, the dose must be diminished.

---

**Iodized Glycerine.**

The *Druggists' Circular* publishes a formula for iodized glycerine, received from a correspondent, who is of opinion that it will be generally preferred to the officinal tincture. It has the advantage of not staining the skin, yet is equally efficacious with the unsightly preparation now in use:

Iodide of potassium, j ℥.
Resublimed iodide, j ℥.
Glycerine, to make, xvj fluid ℥.

Powder the iodide and dissolve in about four ounces of the glycerine; add the iodide and rub well in a mortar until it is dissolved. Lastly, add the remainder of the glycerine.

**Milk of Almonds.**

The *Druggists' Circular* gives the following recipe:—

Blanched sweet almonds, j ℥.
Best glycerine, j ℥.
Distilled water, ½ pint.

Beat the almonds to a smooth paste. Mix the glycerine and water, and then add them very carefully to the paste, triturating all the time.

---

**Diarrhœa Mixture.**

The following is given in the *Philadelphia Medical Times.*

Olei ricini, ♏ xxiv.

Sp. chloroformi, ʒ iss.

Sol. morphia mur., ʒ j.

Pulv. gum. acaciæ, ʒ iiss.

Syrupi, ℥ ss.

Aquæ, ad ℥ iv. M.

A dessert-spoonful every hour and a half until the bowels are quieted.

---

**Method for Making Cod-Liver Oil Palatable.**

Mr. P. L. Simmonds, in the London *Chemist and Druggist,* suggests the following: Take equal parts of ground coffee and bone black; mix them in ten times their combined weight of cod-liver oil, and digest for half an hour at a temperature of about 130° Fahr.; then place the mass on a filter and drain the oil off, and you will have its nauseous taste changed into a pleasant coffee flavor.

---

**Toothache.**

The *Dental Cosmos* gives the following formula:—

Carbolic acid, saturated solution,
Hydrate of chloral, saturated solution,
Camphorated tincture of opium,
Fluid extract of aconite, āā fl. ℥ j.
Oil of peppermint, fl. ℥ ss. M.

Apply by saturating a pledget of cotton (or preferably a small piece of sponge,) and pack closely into the cavity of the decayed tooth.

---

**The Dyspepsia of Smokers.**

Dr. Peters, of Paris, alleges that smoking gives rise to a peculiar form of flatulent dyspepsia, characterized by the fact that in such patients charcoal powder produces no effect. Above all, it is necessary to diminish and forbid the use of tobacco. Pretty good results are obtained from the use of strychnine, especially from the tincture of nux vomica. But these are difficult of administration, and, besides, patients know their names, and fear them. He therefore recommends the following prescription:—

℞

Bitter drops of baume, ʒ j;

Tincture of rhubarb, ʒ v.

Make into forty pills, one or two of which are taken before each meal.

---

**Administration of Croton Chloral Hydrate.**

℞

Croton chloral, gr. xxx.

Glycerinæ, ʒ iss.

Ext. glycyrrhiz., ʒ i.

Aq., et syr. simpl., āā f ℥ iss. M.

Tablespoonful *pro re nata.*

PILLS OF THE ABOVE.

℞
Croton chloral,
Pulv. glycyrrhiz.,
Confect. rosarum, āā gr. xv. M.

Ft. in pil. No. xx.—*Med. Times.*

---

**Antispasmodic Pills.**

The *London Medical Record* gives the following formula:—

Pulv. assafœtidæ,

Pulv. camphoræ, āā ʒ vj.

Ext. belladonnæ, ℈ ii.

Pulv. opii, ℈ i.

Syrupi, q. s.

M. Ft. in pil. No. clxxx.

One to be taken the first day, two the second, and so on until six are taken daily, or two three times a day. Useful in hysterical and spasmodic nervous affections, in connection with bromide of potassium, in doses of ten to fifteen grains.

---

**Chloral Suppositories.**

The production of a chloral suppository containing a sufficient proportion of this drug to cause sleep, has heretofore been deemed impossible. M. H. Mayet, pharmacien, of Paris, has, however, devised the following formula, by which he manages to get forty-five grains of chloral in each suppository:—

℞

Ol. theobromæ, gr. xxx.

Cetacei,

Pulv. chloral, āā gr. xlv.

For one suppository.

These suppositories are of good consistence, and may be easily put into use.—*Medical Times.*

**Peritonitis.**

Patients with simple acute peritonitis, as soon as admitted, were put under the influence of opium, and kept so until the acute symptoms subsided. Warm fomentations were applied to the abdomen, such as warm turpentine cloths wrung from hot water, and very light poultices. A liquid diet of milk punch, beef-tea soup, and such articles only, was allowed. During convalescence, if tonics or laxitives were required, they were used.—*N. Y. Hosp. Rep.*

**Elastic Ligature.**

Several cases of fistula in ano have been treated here of late, by means of the elastic ligature, and the patients have suffered but little inconvenience while the ligature was cutting through. They were up and about, most of the time, and suffered but little pain. In one case, the ligature cut its way through in five days; in two other cases, in seven days, and in every case a fine granulating surface was left behind, which healed much more readily and satisfactorily than the wound made by the knife.—*N. Y. Hosp. Rep.*

**Diarrhœa.**

Simple diarrhœa was treated by astringents of various characters. The most simple and frequently used was chalk mixture, or this in combination with confection of opium and tincture of kino krameria. Subnitrate of bismuth and tannic acid were also used, the latter combined with simple or camphorated tincture of opium. After the diarrhœa was stopped, the diet was regulated for some time, consisting mostly of bland and easily digested articles. If any debility remained, tonics were given until it was removed.—*N. Y. Hosp. Rep.*

**Plugging the Posterior Nares.**

This operation is one of the bugbears in surgery, therefore a simple method of performing it may be repeated without giving offence. It may not be anything new, but the ease with which it was performed in this case was certainly agreeable to the patient and pleasing to the surgeon. A small bougie, having bulbous extremity (a fine catheter may answer the same purpose) was introduced into the nostril to be plugged, and carried along the floor until it could be seen emerging into the pharynx; this extremity was then seized with a pair of long forceps—which can usually be done without inconvenience to the patient—and brought forward through the mouth. A strong silken cord was then attached to it, and to the other extremity of the cord was fastened a pledget of lint. The bougie was then withdrawn from the nostril, and with it the string to which was attached the plug, and then this was drawn through the mouth, and quickly carried up behind the velum into the cavity for which it was designed. The operation gave the patient but little inconvenience, and the plug was to remain until the *natural secretion of the parts loosened it* sufficiently to permit a spontaneous removal.

**Antidote for Arsenic.**

In the *German Pharmacopœia* an "Arsenic Antidote" is ordered. It is to be prepared thus:

Solution of persulphate of soda, 60 parts.
Water, 120 parts.
Mix, and add
Calcined magnesia, 7 parts.
previously rubbed with
Water, 120 parts.

These are to be shaken together until a soft regular mass is obtained. The solution of persulphate of iron is prepared by dissolving 40 parts of ferri sulph. pur. in 40 parts of aq. dist. Add acid sulph. pur., 7 parts; boil in a porcelain capsule; then add very gradually acid nitric pur., 12 parts, or until the solution does not decolorize a solution of permanganate of potassium. Evaporate to a solid mass, which dissolve in 40 parts of distilled water.

**Tincture of Arnica a Dangerous Application.**

Dr. James C. White has an article in the *Boston Med. and Surg. Journal*, for January 21st, 1875, on the poisonous action of tincture of arnica on the skin. He relates three cases where its use as a lotion for bruises occasioned severe attacks of acute eczema. Dr. White is inclined to believe that such results are more common than is generally known. The use of arnica as a household remedy is exceedingly extensive, and the reason that the poisonous effects often produced upon the skin do not lead to its disuse, is that the true nature of these effects are seldom recognized, but are attributed to the original injury for which the arnica is applied. Whatever beneficial results may follow the use of that tincture Dr. White would ascribe wholly to the alcohol. Hebra has long ago entered a protest against the use of this supposed remedy, and its irritant properties are described by Tilbury Fox. Dr. White thinks the profession should cease to accord to so useless and dangerous a drug a confidence to which it is in no way entitled.

**Rheumatism.**

It has been said that rheumatism is quite apt to follow in the wake of some chest trouble, such as asthma, phthisis, etc., and there were several cases in the wards which seemed to illustrate this idea. They were, for the most part, cases of phthisis, and the prescription in use was denominated that for "rheumatism of phthisis." One or two or more joints became more or less swollen, stiff and inflamed, and the patients were placed upon:—

℞

Potass. iodid, ℨ i;

Fld. ext. conium, ℨ iij;

Tr. opii. camph., ℥ ij;

Aq. aurant. flor., ℨ iv;

Aquæ, ℥ iv. M.

S. Teaspoonful t. i. d.

---

**Diarrhœa of Phthisis.**

AN excellent article of diet when this symptom is present, is milk boiled with mutton suet until it is thick as cream. The method of preparing it is to put a piece of suet into a bag and boil it in the milk until the required consistency is obtained. To this may be added such remedies as the physician may wish to administer, as persulphate of iron, opium, belladonna, etc. Belladonna is said to be very serviceable, for the reason that it possesses the power of producing contractions in the unstriped muscular fibre of the intestines. The following pill was also recommended:

℞

Resin of turpentine, gr. iij;

Nitrate of silver, gr. ¼;

Opium, gr. ¼. M.

S. One P. R. N. Any of the oleo resins, perhaps, may be used.

---

**Sore Nipples.**

Julius Fehr, M. D., writes to the *Medical Record* as follows: "The curative as well as the palliative treatment of sore or cracked nipples being well known to be futile, my aim for a long time was directed to the finding of a *reliable prophylactic*. After trying a great many formulæ of others, and combinations of my own, I came at last to the use of *tannate of lead*, the "cataplasma ad decupitum" of the Pharmacopæia Germanica, with the addition of a little glycerine to modify, in some degree, the excessive drying properties of that preparation. This "*plumbum tannicum pultiforme*," I had applied, for about one month before parturition, two or three times a day, directly to the nipples. This, I found "TANNED" the nipples in so thorough a manner that they were perfectly able to withstand all suckling and all pulling, on the part of the infant, successfully. At the same time, I used a piece of *cotton felt*, about one and a half inches in diameter, and half an inch in thickness, with an aperture in the middle large enough to give free access to the nipples. This will not only prevent the pressure of the garments on the nipples, but will give, at the same time, to the nipples a chance to develop themselves better, which is often so much needed.

---

**Compressed Sponge for Abscess.**

A patient had been suffering from mammary abscess for three weeks, but without any special benefit from treatment in checking the discharge of pus. It was decided to try the effect of compressed sponge, and for this purpose a sponge about ten inches in diameter was subjected to pressure and then applied by means of a bandage over the breast. After it had been in use forty-eight hours, the abscess was completely cured. No pain was experienced by the patient, and in this case the opening in the breast was three inches above the dependant part of the abscess. In applying a sponge to the breast in this class of cases, it is found of advantage to compress it when dry. After it is applied to the breast and firmly secured in position, a little water is poured upon it to cause expansion and the necessary pressure.—*Med. Times.*

---

**Croton-Chloral in Diseases of Children as Compared with Chloral-Hydrate.**

Bouchut (*Centralbl.;* from *Gaz. Med. des Hopitaux,*) is inclined, from his experience with croton-chloral, to attribute to this drug only trifling results, compared with those obtained from chloral-hydrate. While the latter in doses of forty to sixty grains will produce such perfect anæsthesia that abscesses may be opened and teeth extracted without pain, croton-chloral, even in drachm doses, scarcely causes sound sleep. The single advantage possessed by croton-chloral is its more agreeable taste. Bouchut has compared both remedies in chorea of children, and gives chloral-hydrate decidedly the preference. (According to Liebreich, croton-chloral, the taste of which is intensely bitter, acts chiefly upon the nerves of the head. In a solution in water of two scruples to six ounces, given in dessertspoonful doses every two hours, stubborn hyperæsthesia of the trigeminus has been cured in many cases and relieved in others.)

**Liquor Ferri Perchloridi as a Local Application in Cancerous Ulceration of the Uterus.**

When the course of the cancer is rapid, the application of the strong perchloride of iron is of little use; but when the disease is purely epithelial, or chronic, or rodent in character, it relieves, or sometimes seems to cure bad cases. Its application rarely causes pain. All discharges should be washed away from the cancer, and the breech of the patient raised to prevent any overflow of the solution over the vulva. Cotton-wool saturated with the perchloride is then applied, and any superabundant solution which a slight pressure of the wool causes to flow out, is to be sucked up by a sponge from the bottom of the vagina. The wool is to be retained in its place by a loose plug of tow in the vagina, and the vulva oiled before the patient rises from the couch.—*The Lancet.*

**Typhoid Fever.**

Dr. George Johnson, in the London *Practitioner*, takes the ground that in the treatment of typhoid fever carefnl nursing and feeding are of primary importance, while, as a rule, no medicines of any kind are required, and when not required they are often worse than useless. Diarrhœa is a less frequent symptom than before this plan was adopted, and when it does occur it is far more tractable, while tympanitic distension of the abdomen is a rare event. The mischievous opiate enemata and the torturing turpentine stupes have disappeared together. He believes that one of the main reasons why there is less diarrhœa than formerly is the careful abstinence from the employment of irritating drugs of all kinds. As a rule, a fever patient at "King's" has the "yellow mixture," which is simply colored water; and, except an occasional dose of chloral to produce sleep, and a tonic during convalescence, no active medicines of any kind. These patients are fed mainly with milk, with the addition of beef-tea and two raw eggs in the twenty-four hours, and wine or brandy in quantities varying according to the urgency of the symptoms of exhaustion, especially in the advanced stages of the disease; but in many of the milder cases, and especially in the case of children, no alcoholic stimulants are required from the beginning to the end of the fever, and when not required, they are, of course, says Dr. Johnson, best withheld. He gives no irritating drugs of any kind, and has no doubt that the comparative infrequency of severe and obstinate diarrhœa amongst his enteric fever patients during the last few years is partly attributable to the discontinuance of mineral acid treatment.

**Diarrhœa.**

In a paper in Virchow's *Archiv*, Dr. Hartsen observes that diarrhœa of all sorts goes along with an irritable state of the intestinal canal, and any increase of this irritability is to be carefully avoided. He considers that the more usual astringents are in addition, irritants; and he instances among them the salts of lead, zinc, and bismuth. In all cases, soothing means should first be adopted; and of these, warm applications to the abdomen, in the form of bread poultices, or fomentations, are perhaps the best. The chief medicine recommended is opium, which soothes, but in large doses interferes with digestion. If the diarrhœa be so violent as to hinder the absorption of opium introduced into the stomach, then morphia should be injected subcutaneously. Of equal importance is the diet. If the person be strong, everything, both solid and fluid, should be withheld; but, where this cannot be done, the food should be of the lightest and simplest. The author especially refers to rice and arrowroot as simple vegetable diets, while any animal food given should be free from fat. Milk should not be too much used, and in any case should be boiled.

**Bronchial Catarrh and Winter Cough.**

In a note sent to the *British Medical Journal*, Drs. Sidney Ringer and Wm. Murrill state that in the treatment of these complaints they have employed tar in two-grain doses, made into a pill, every three or four hours. From October to January, inclusive, its effects were watched on twenty-five patients, whose ages varied from thirty-four to seventy. All these patients had suffered several years from winter cough during the whole winter.

Each attack of the paroxysmal and violent cough lasted from two to ten minutes, recurring ten or twelve times in the day and breaking their rest at night. Expectoration was abundant, frothy, and purulent. Breathing was short on exertion, but most could lie down at night without propping. The physical signs showed a variable amount of emphysema, with sonorous and sibilant rhonchus, and occasionally a little bubbling rhonchus at the base. These patients usually began to improve from the fourth to the seventh day; the improvement rapidly increased, and in about three weeks they were well enough to be discharged. The improvement was so decided that even those patients who, in previous years, had been confined to the house during the whole winter, returned to their work. On discontinuing the tar, relapses often occurred in a week or two, but on re-administering the medicine relief was again obtained.

**Diabetes Millitus.**

Mary G——, of Plasket, aged seventeen, who has never menstruated, came to the dispensary on January 14th, 1874. Though previously healthy, for the last six weeks she has gradually become weak and inert. Her skin was harsh and dry, and her appetite voracious. There was great constipation, thirst, and polyuria. She is a nervous subject, but there was no history of a fright or change of diet. The urine (sent that day week) showed much sugar, by Trommer's test. She was given fifteen drops of tincture of perchloride of iron three times a day, and skim-milk ordered. For the next fortnight she steadily got worse, and then the treatment was changed to ten drops of tincture of opium, and a week later, fifteen drops, three times a day, with croton-oil pills. By this time she was so weak that she could not come to the dispensary herself. On February 18th, a sixth of a grain of nitrate of uranium, in water, was given three times a day, and gradually raised to the third of a grain. A week later she was much better. The week following, the bowels were regular, and the appetite and the quantity of urine no longer excessive; while on March 4th, and for a fortnight after, she had gone back to her usual diet, and felt nothing wrong with herself save some muscular weakness.—*Physician and Pharmacist.*

---

**Treatment of Fever.**

In an article in the *Dublin Medical Journal*, Dr. Jones reaches the following conclusions:—

1. That in the treatment of fever, typhus and other forms, too much reliance has been placed on *alcoholic* stimulants, and that fashion, rather than reason, has swayed many in their *indiscriminate* employment.

2. That the percentage of cases requiring such stimulants is a low one; and that while our administration of them, as regards quantity and kind, must depend entirely on the condition of the patient, still the utmost caution (with our present knowledge of their physiological action) is required.

3. That in digitalis we have a powerful cardiac stimulant, which, while it gives force to the heart, does not do so at the expense of the system, but rather is a conservative agent, which controls expenditure and limits waste of vital action; always, of course, remembering that a large number of cases will recover without any specific treatment save that care and guidance which provides for the wants of the system and secures the patient from the risks of complications. That digitalis appears to be indicated in the early periods of many cases of typhus in which we have a rapid pulse and high temperature range, regulating our administration by its effects on both, using it rather with the object of guiding the patient up to a certain point than of curing the disease.

---

**Insulation of the Bed in Rheumatism.**

Our readers will recollect Dr. Wagenhals's report on this subject, of which an abstract was given in the *Journal* for May, 1875. Dr. E. Playter, of Toronto, gives a case of the kind in the *Canada Lancet.* The patient was ordered to be insulated, which was done by placing the legs of the bedstead in four glass salt-cellars. The next day he was decidedly easier, and continued to improve rapidly, so that he was entirely free from pain in three or four days more, and able to walk about the house.

The constitutional symptoms in this case were not of a very marked character. The heart's action was labored and irregular in the beginning, but not quickened, tongue slightly coated, white, with considerable thirst, but perspiration not profuse; though these symptoms, excepting the heart's action, were gradually becoming more aggravated up to the time when insulation was tried.

The patient had had two previous attacks, similar, but of much greater duration; from which he had entirely recovered. His father had suffered from two or three very severe attacks of acute rheumatism.

Dr. Playter adds, "One such case does not prove much in favor of insulation, but the improvement in the one above referred to commenced earlier, was more rapid aud decided than that of any other case of like severity which I have treated, in a practice of about fifteen years."

---

**Biliary Calculi.**

At a late meeting of the Societe de Biologie, Dr. Laborde gave the results of his researches on "Biliary Calculi; why they are painful, and how they should be treated." The secreting bile-passages, he said, were contractile, and became affected with spasms under the influence of direct or indirect excitement. Their contractility was like that of the smooth muscular fibres of organic life, the existence of which in the walls of the bile-channels had been shown by histological researches and physiological experimentation. The mucous membrane of the passages was possessed of great sensitiveness, which manifested itself under the influence of exciting causes, both by intense pain and by reflex action, as shown in the spasm of the biliary canals. These phenomena were especially determined by the presence of, and contact with, foreign bodies (biliary calculi,) the spontaneous migration of which was rendered difficult by these circumstances, and, when affected, would occur only after some time, with this particularity, that the foreign bodies might recede even into the biliary vesicle. Morphia and hydrate of chloral administered simultaneously formed the best mode of treatment. They exerted both an anæsthetic and paralyzing influence, with the effect of producing cessation of the spasmodic state, distension of the passages, and accumulation of the biliary fluid, which acted on the foreign body by a kind *vis a tergo* and forced it down the intestines.—*Monthly Abstract of Medical Sciences.*

**Hysteria.**

Hysterical patients were given one of the bromides in full medicinal doses; if this did not exercise a controlling influence over the paroxysms, then some one of the antispasmodics, valerian, assafœtida, or compound spirits of ether, were tried. If the patient was anæmic, tonics and a liberal diet were given. All menstrual derangements were given close attention. If the patient suffered from suppressio mensium, every effort was made to establish that function by tonics, stimulating purgatives, aloetic or otherwise, hot hip baths at every revolution of the menstrual period, and cold to the spine. The following mixtures, combining antispasmodic and purgative properties, well adapted to constipation when occuring in this disease, were used in many of the cases:—

℞

Infusi valerianæ, f. ℥ ij;

Tincturæ hyoscyami, f. ʒ ij;

Extracti sennæ fluidi, f. ʒ ij;

Tincturæ rhei, f. ℥ ss.

Ft. sol.

Sig.—A tablespoonful every three hours.

℞

Ext. sennæ fluid, f. ℥ ss;
Spt. æth. chloric, f. ℥ ij;
Liq. ammonia acetatis, f. ℥ j;
Aquæ camphoræ, f. ℥ ij;
Syrupi auranti corticis, f. ℥ ss.
Ft. sol.

Sig.—A tablespoonful every three hours.—*N. Y. Hosp. Rep.*

**Delirium Tremens.**

In those cases of the disease presenting marked hallucinations, and even delirium, the patient was given large doses of the bromides, twenty to thirty grains, three times per day. In other cases, opiates, subnitrate of bismuth, and small quantities of alcohol, in some palatable form, were used, instead of the bromides. An infusion of hops was used in large doses, and with some advantage. Under its use, the patient became calm, less restless, and inclined to sleep. As a stimulant to the alimentary mucous membrane, pills of capsicum, of five or eight grains, and aromatic sulphuric acid, in doses of fifteen drops, three times per day, were used. Where the attack was dependent upon a severe and recent debauch, the treatment varied somewhat from the above. As the chief symptom to be counteracted here was cerebral congestion, purgatives and the bromides were preferable to opium. The following is a favorite purgative mixture of one of the staff:—

℞

Sodæ et potassæ tartratis, ʒ iij;

Spiriti ætheri nitrosi, f. ʒ iss;

Extracti sennæ fluidi,

Extracti rhei fluidi, āā f. ʒ ij;

Tincturi hyoscyami, f. ʒ ij;

Aquæ menthæ piperitæ, f. ℥ iij.

Ft. sol.

Sig.—A tablespoonful every two hours.

The food was chiefly liquid, consisting of soups, milk, beef essence, etc. During convalescence, the patient's general condition was improved by tonics, and as much good food as he could thoroughly digest.—*N. Y. Hosp. Rep.*

**Dysentery--Chronic and Acute.**

In acute sporadic dysentery, the patient was put at rest in bed, and given some mild purgative, either castor oil or one of the salines. After the bowels had been evacuated, a full dose of some opiate preparation was given to restrain the evacuations, relieve the tenesmus, and procure rest. If this did not suffice, the saline was repeated and another dose of opium given. If the patient desired it, small fragments of ice were allowed him, at liberty. Injections of cold water into the rectum, though at first painful, in a short time afforded great relief, decreasing the number of evacuations, and to a large degree removing the tenesmus. If, after checking the dysentery, there remained any debility, it was removed by tonics, healthy exercise, and a good diet. Those patients suffering from a chronic form of dysentery were generally much debilitated when they came under observation, and consequently required a sustaining treatment. They received a liquid but very nutritious diet, and tonics, if they were required. For the local disease of the bowel, various agents and methods were resorted to. A pill of opium and nitrate of silver, containing a quarter of a grain of each, was given, in most cases, for two weeks or more, but we cannot recall a single instance where its use produced any decided benefit. A powder of subnitrate of bismuth and pepsin, containing ten grains of the former to five of the later, were often used, giving three or four per day, according to the requirements of the case. Suppositories of opium were used. They checked the evacuations for the time, and often for some time after their administration. More attention was given to improvement of the patient's general health than to local measures.—*N. Y. Hosp. R.*

---

**Chloral-Hydrate as an Antidote to Strychnia.**

A most extraordinary case of recovery from strychnia poisoning, due to treatment by chloral-hydrate, is reported in last week's *Lancet* by Dr. Charteris, physician to the Royal Infirmary, Glasgow. On March 12, a butcher of that city, in a despondent condition, bought from a chemist two sixpenny packets of Gibson's Vermin Killer. These contained two grains of strychnia in each. Then he adjourned to the parlor of a public-house, bought some whiskey, in which he dissolved the contents of the two packets, added some ginger ale, and drank off the mixture. In order to make sure work of it he bought another draught of ginger ale, and drank it from the same glass, so as to dissolve any remnant of the poison. This was about 11.30 A. M. Then he walked across the street to a butcher's shop and asked to be allowed to sit down. A succession of tetanic fits ensued, and these seem to have continued for several hours. About 1 P. M. an emetic of hot water and sulphate of zinc was given, which, however, produced only partial vomiting. It was 3.30 P. M. when he was brought to the Royal Infirmary. He was in great agony: violent tetanic spasms followed each other in quick succession, increasing in severity. With great difficulty, the stomach-pump was passed, but only some sour watery fluid was withdrawn. At 4.50 P. M. 10 grains of chloral-hydrate in syrup were administered. Twenty minutes after, the dose was repeated, and at 5.30, 20 grains were given. Immediately afterwards there was a severe and prolonged spasm, succeeded by a flaccid state of the muscular system, hurried respiration, quickened pulse, and drooping eyelids—phenomena which indicate the effect of chloral. Three more doses, 10 grains each, were administered between this and 9.30 P. M., the symptoms of poisoning subsiding. The patient was dieted on milk and rice, and in four days had completely recovered. He said if he wished to die again he would choose something other than Gibson's Vermin Killer. Dr. Charteris afterwards tried some experiments on rabbits, and found that the administration of chloral-hydrate after strychnine most decidedly checked the poisonous effects, though in one case where a longer interval had been allowed to elapse between the poison and the antidote, the animal was paralyzed from the middle of the spine downwards. It is stated, also, to account for the slowness of the action of the strychnine in the man's case, that he had previously taken a substantial breakfast of ham and eggs.

---

**The State of the Pupil in Chloroform Anæsthesia as an Indication of Danger.**

M. Budin, interne of the hospitals, in examining with care all the details of chloroformization, with the end of discovering some sign which may guide the surgeon in its administration, has observed that there exists a certain relation between the state of the pupils and the more or less profound anæsthesia of the subject.

1st. There exists in the surgical anæsthesia produced by chloroform, a constant relation between the state of the pupil and the period of anæsthesia.

2nd. During the period of excitation the pupil is dilated.

3rd. The period past, the pupil contracts; its atresia, very marked for several minutes, accompanies, in general, complete anæsthesia.

4th. Dilatation of the pupil supervening during an operation, indicates, in general, that anæsthesia is less profound, and that return to sensation is near.

5. The state of the pupil may then serve as a guide in the administration of chloroform.

6th· During an operation of long duration, if the patient is to be kept insensible and immobile, the pupils must be kept constantly contracted.

7th. Finally, efforts at vomiting may produce dilatation of the pupils, dissipate the insensibility, and awaken the patient; they annihilate, in part, the effects of anæsthesia.

---

**Gastro Enteritis.**

To restrain the vomiting that was often persistent, the patient was placed at rest in the recumbent posture and given small fragments of ice, and directed to swallow them undissolved. If this does not restrain it, other agents were resorted to, such as subnitrate of bismuth and tannic acid, in powder, camphor water, oxalate of cerium and chalk mixture. Little or no food was allowed by the mouth, the patient being sustained by nutritious enemas; by this means the stomach was placed entirely at rest, and all sources of irritation removed. To prevent purging: from the enteric complication, the patient was first given a small dose of castor oil, to remove all feculant matter from the bowel, and then a dose of opium, combined, or not, with some astringent agent calculated to restrain their action. The following formulæ were very much used:

℞

Confectionis opii., gr. xxxvj;

Misturæ cretæ, f. ℥ ij;

Tincture kino., f. ʒ iij;

Ft. sol.

Sig.—A tablespoonful three times per day.

℞
Aquæ camphoris, f. ℥ ij;
Morphiæ acetatis, gr. ij;
Acidi tannici, gr. x;
Ft. sol.
Sig.—A teaspoonful every two hours.

In several cases, a blister was applied over the small intestines; in two instances it produced a marked decrease of all the symptoms, the pain subsiding to a great extent, and likewise the griping.—*N. Y. Hosp. Rep.*

---

**Tapeworm.**

At a meeting of the Berlin Medical Society, held October 28th, 1874, Herr Pincus remarked, that many medical men consider a preparatory treatment necessary before administering anthelmintics. This preparation consists of oils and low diet for some days beforehand, or of two or three days physicking, which irritates the worms. The consequence, he states, is that when the anthelmintic is given, portions only of the body, and not the head, are expelled. The remedies may be divided into two classes, namely, those which paralyze the movements of the proglottides, kousso, panna [the rhizome of *Lastrea* (*Aspidium*) *athamanticum*, much esteemed by the Zulu Caffres, and somewhat resembling our male-fern,] kameela; and those which act upon the organs of adhesion, as the bark of pomegranate-root and male-fern. Whichever be employed, the best result is obtained by giving a full dose without any preparatory medication. Again, it has been advised, when the worm is found to protrude from the anus, to do nothing except let the patient sit over warm water; this may be done for hours, and the only consequence is, that after from eight to ten feet of worm have come away, there is no further progress; probably because the head is not paralyzed, and remains adherent, whilst the proglottides come away. In such cases Dr. Pincus gives a narcotic clyster, if the movements of the worm be lively; and then, in less than an hour, even when the tapeworm is broken, he has found the head to come away. The older remedies, such as pomegranate-root bark, are preferable, because they do not much derange the patient's health. Professor Henoch remarked that narcotic clysters, even with chloroform, had been recommended some time since. He asked if Dr. Pincus had himself experimented upon the worms. Dr. Pincus said that he meant substances which narcotized the worms themselves, particularly pomegranate-root bark. He had made experiments upon the proglottides passed by patients; the movements of these ceased immediately when subjected to kousso, panna, or kameela. He had made no direct experiments on the action of the other remedies in paralyzing the adhesive organs. Professor Liebreich drew attention to the koussine or koussein prepared by Bedal, of Munich, which was not an alkaloid, but contained the resin of kousso. Two grammes of this preparation have the same effect as twenty grammes of kousso [ʒ ss koussine is equal to ʒ v of kousso.] Herr Paasch had also used pomegranate-root bark, but considered no cure as complete unless the worm came away *sua sponte*. Professor Henoch denied that the passing of proglottides denoted the worms being themselves out of health; the proglottides severed themselves when sexually ripe, in order to pursue their further transmigrations. Herr Furstenheim mentioned the thick lozenges prescribed by Rosenthal as very convenient in practice. They contained kousso. Her Pincus believed that the proglottides become separated when the fibres which connect them are so softened and worn that the alkaline mucus of the bowel dissolves them.—*Berliner Klinische Wochenschrift.*

---

**Chronic Bronchitis.**

In the reports of treatment adopted in the Roosevelt Hospital, New York, Dr. Heineman prescribes as follows:

℞
Oleum lini, ℥ ij;
Mucilagi acacie, ℥ ijss;
Syrup tolu, ℥ j;
Ol. terebinth., ℥ iij;
Aquæ cinnamomi, ad. ℥ viij. M.
Sig: ℥ ss. t. i. d.

**Examination of Urine.**

Medical practitioners are beginning to realize the great aid which the examination of this fluid gives them in diagnosing disease. The pharmaceutist is called upon more frequently to make these examinations for physicians, and as an aid to pharmaceutical students, the writer recently gave lectures upon the subject in the summer course at the College of Pharmacy in this city. It is not proposed to give the minutiæ of the lectures to our readers, but to present, with a brief introduction, the scheme for a chemical examination of urine, as given on the screens used in the lectures.

In following the scheme proposed, it may be said that usually it is unnecessary to make all the experments, as some of the abnormal ingredients are quite infrequent.

The general examinations desired are for albumen, sugar, phosphates and urea, the specific gravity and its action on litmus paper.

In testing, take a small portion of the filtered urine for each separate test, and note at once the reactions, reserving each until the examination is complete.

While the daily quantity usually voided by an adult rarely exceeds fifty fluid ounces, yet there are occasions when this limit is greatly exceeded. In one case known personally to the writer, no less than *nine gallons* were passed as a daily average for over thirty days, and the patient now passes daily about *one and a half gallons.* This has continued for over six years, and has not kept the patient in the house, but on the contrary the person has, during most of the time, been occupied with regular daily duties.

The scheme which follows is only intended to enable the experimenter to recognize the several normal or abnormal constituents usually sought for, and this is copied almost verbatim from the lecture screen.

The use of the microscope in the hands of a careful experimenter is of wonderful assistance, but cannot be enlarged upon in this connection. To determine the amount of some of the constituents, we will again give some tests for this purpose.

Morbid urine may contain (1) too great or too small a proportion of one or more of its normal constituents. These are *Urea, Uric Acid, Urates, Phosphates, Chlorides;* (2) or it may contain one or more ingredients not usually found in healthy urine: *Albumen, Sugar, Blood, Pus, Bile, Chyle.*

CHEMICAL EXAMINATION OF A PORTION OF (FILTERED) URINE.

1. Acid or alkaline; use litmus paper as a test.
2. Specific gravity; use sp. gr. bottle or hydrometer.
3. Nitric acid is added; if crystals form abundantly there is excess of UREA; if a precipitate, ALBUMEN may be present.
4. A portion is heated, a *precipitate* indicates either ALBUMEN or PHOSPHATES.
5. To the precipitate from No. 4 (in the tube) add nitric acid. PHOSPHATES *are dissolved*, ALBUMEN is *not dissolved.*
6. To a portion of urine add the copper test solution with heat; a reddish-yellow precipitate indicates SUGAR.
7. A portion is tested with nitric acid; if precipitate is abundant and of light color—ALBUMEN; if scanty and of red color—URIC ACID.
8. The urine when boiled forms a dark coagulum—BLOOD.
9. The urine mixed with a warm solution of urate of ammonia gives a *pink precipitate*; PURPURINE.
10. The urine (deprived of albumen by heat) has a few grains of sugar added, and then sulphuric acid drop by drop; a deep red color indicates BILE.
11. Solution nitrate of silver precipitates CHLORIDES. (The urine must first be freed from Albumen. Each grain of precipitated chloride of silver, when dried at 212° F., represents $\frac{4}{10}$ gr. chlorides.)
12. Unfiltered urine, if milk-colored, or, if on standing, it gives a heavy milk-white deposit, contains CHYLE.

EXAMINATION OF URINARY DEPOSITS.

1. Deposit white, see 2; colored, see 6, 7, and 8.
2. Deposit soluble when heated; URATES.
3. Deposit soluble in ammonia; CYSTIN.
4. Deposit soluble in acetic acid; EARTHY PHOSPHATES.
5. Deposit insoluble in acetic acid; OXALATE OF LIME.
6. Deposit chrystalline; URIC ACID.
7. Deposit amorphous, faintly colored, readily soluble when heated; URATES.
8. Deposit strongly colored, slowly soluble when heated; URATES tinged with PURPURINE.
9. Deposit greenish-yellow, easily diffused by agitation; PUS.
10. Deposit ropy and tenacious; MUCUS.—*P. W. B., in Druggists' Circular.*

